# Functional Integration of Different‐Sex Gonad Transplants Into the Adult Mouse Hypothalamic Pituitary Gonadal Axis

**DOI:** 10.1002/adbi.202500316

**Published:** 2025-12-07

**Authors:** Daniel R. Pfau, Monica A. Rionda, Evelyn Cho, Jamison G. Clark, Robin E. Kruger, Ruth K. Chan‐Sui, Vasantha Padmanabhan, Molly B. Moravek, Ariella Shikanov

**Affiliations:** ^1^ Obstetrics & Gynecology University of Michigan Ann Arbor Michigan USA; ^2^ Pediatric Endocrinology University of Michigan Ann Arbor Michigan USA; ^3^ Biomedical Engineering University of Michigan Ann Arbor Michigan USA; ^4^ School of Social Work University of Michigan Ann Arbor Michigan USA; ^5^ Molecular and Integrative Physiology University of Michigan Ann Arbor Michigan USA; ^6^ Obstetrics Gynecology and Reproductive Biology Michigan State University East Lansing Michigan USA; ^7^ Reproductive Endocrinology and Infertility Department of Women's Heath Henry Ford Health Detroit Michigan USA; ^8^ Cellular and Molecular Biology Department University of Michigan Ann Arbor Michigan USA

**Keywords:** gender‐affirming hormone therapy, ovary, sex difference, testis, transgender

## Abstract

Gender‐affirming hormone therapy (GAHT) relies on exogenous hormones to produce hormonal milieus that achieve and/or maintain embodiment goals. Another potential route to these endpoints is transplantation of novel steroidogenic tissue. To develop a pre‐clinical model, we asked whether different‐sex gonad transplants can be functionally integrated into the adult mouse hypothalamic‐pituitary‐gonadal (HPG) axis. Adult male and female mice are gonadectomized and implanted with gonads from genetically matched but different‐sex pups. Controls received gonads from same‐sex pups. Temporal changes to gonadotropin and steroid hormone levels reveal the decoupling of the HPG following gonadectomy and gonad‐dependent levels after transplanting donor gonads. After six weeks, histological structures in transplanted gonads are consistent with expected steroidogenesis and gametogenesis. Interestingly, pituitary, ARC, and AVPV mRNA showed gonad‐ and sex‐dependent expression patterns. Future work with this technique could lead to translation to gender affirming care and explorations of gonad‐dependent sex differences in biomedical and basic research.

## Graphical abstract

This study introduces and investigates different‐sex gonad transplants in a mouse model of gender‐affirming hormone therapy. Ovaries and testes implanted into gonadectomized adults exhibit gametogenesis and steroidogenesis independent of the sex of the host. Gonad‐dependent neurotransmitter and steroid hormone receptor mRNA expression in the hypothalamus and pituitary may facilitate this surprising plasticity in adulthood.

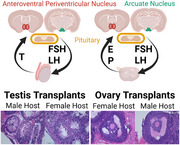

## 1 Introduction

Many transgender, non‐binary, and gender diverse (TNG) individuals utilize GAHT to produce hormonal milieus that achieve and maintain embodiment goals [1]. Individuals may take testosterone (T) to increase circulating levels and/or suppress ovarian function, or estrogen (E), progesterone, and/or anti‐androgens (collectively termed E‐GAHT) to increase their levels and suppress testicular function and androgen action [2, 3]. Attaining physical results that cannot be generated by GAHT may involve gender‐affirming surgeries [4]. While gonadectomies may remove the need to suppress gonadal function, continued GAHT use is required to maintain optimal steroid hormone levels for overall health [2]. The range of GAHT regimens and surgical procedures reflects variation in individuals’ responses to treatments and diverse patient needs [5, 6, 7]. As these needs are better understood [7, 8], novel interventions may increase the number of desirable phenotypes gender‐affirming treatments can offer [9, 10]. One potential surgical intervention could involve transplantation of different‐sex donor gonads (see ‘*Sex nomenclature*’ in Materials & Methods for definition of “different‐sex” and “same‐sex”), which would rely on plasticity of hypothalamic and pituitary function to stimulate endogenous hormone production from transplanted gonads. Endocrine function is routinely restored after thyroidectomies, when parathyroid glands may be removed with the thyroid. Parathyroid function can be replaced by implanting parathyroid tissue subcutaneously or intramuscularly in the forearm [11, 12]. Critically, the success of transplanted parathyroid glandular tissue does not rely on microvascular anastomosis. Similarly, ovarian tissue placed in the forearm without anastomosis survives and functions [13]. Unlike GAHT, this procedure would reintroduce gonad cycles and homeostatic control of steroidogenesis, which may improve patient outcomes. For example, gonadectomy followed by T‐GAHT is detrimental to bone mineral density measures for female mice, but providing low‐dose E, to mimic the low levels of circulating E produced by the testes, can rescue musculoskeletal architecture [14]. Transplanted testicular tissue would automatically provide low levels of E. Further, non‐steroidal gonadal products are not given as part of GAHT regimes [6], so another benefit of gonad transplants over GAHT would be the replacement of protein‐based signals, like inhibins and activin, particularly for patients who have received gonadectomies. Hypothetically, the results achieved with pharmacological T‐GAHT and E‐GAHT could be reproduced and/or improved by endogenous testicular and ovarian steroidogenesis, respectively.

It has been well‐established in humans and animal models that transplanted autologous ovarian tissue, removed for fertility preservation prior to gonadotoxic cancer therapies, restored ovarian endocrine function, demonstrated by elevated levels of ovarian hormones, regular menses, and even live births [15, 16, 17, 18]. Though relatively less studied, autologous transplants of testicular tissue or cells also restore hormone function in non‐human primates [19, 20] and survived up to 6 months in the only human case study [21]. For these transplants, same‐sex gonadal tissue integrates into the hosts’ hypothalamic‐pituitary axis by responding to circulating gonadotropins and secreting hormones to reinstate the negative and positive feedback necessary for steroid hormone homeostasis and gonad function. Integration of transplanted different‐sex gonads into the HPG axis of the host requires these three tissues to communicate in a novel way. Though hypothalamic and pituitary sex differences enable the unique functions of each gonad [22, 23, 24, 25], the hypothalamus and pituitary of humans respond to circulating T and E levels regardless of an individual's sex [26, 27, 28, 29]. Further, ovarian tissue transplanted from female into male rhesus monkeys led to pre‐ovulatory gonadotropin surges and cyclic gonadotropin and steroid hormone release [30]. Unlike ovaries, testicular transplants into different‐sex non‐human primates have not been performed. It is currently unknown whether human hypothalamic and pituitary plasticity extends to maintaining steroid hormone homeostasis and cycles for different‐sex gonads.

**FIGURE 1:**
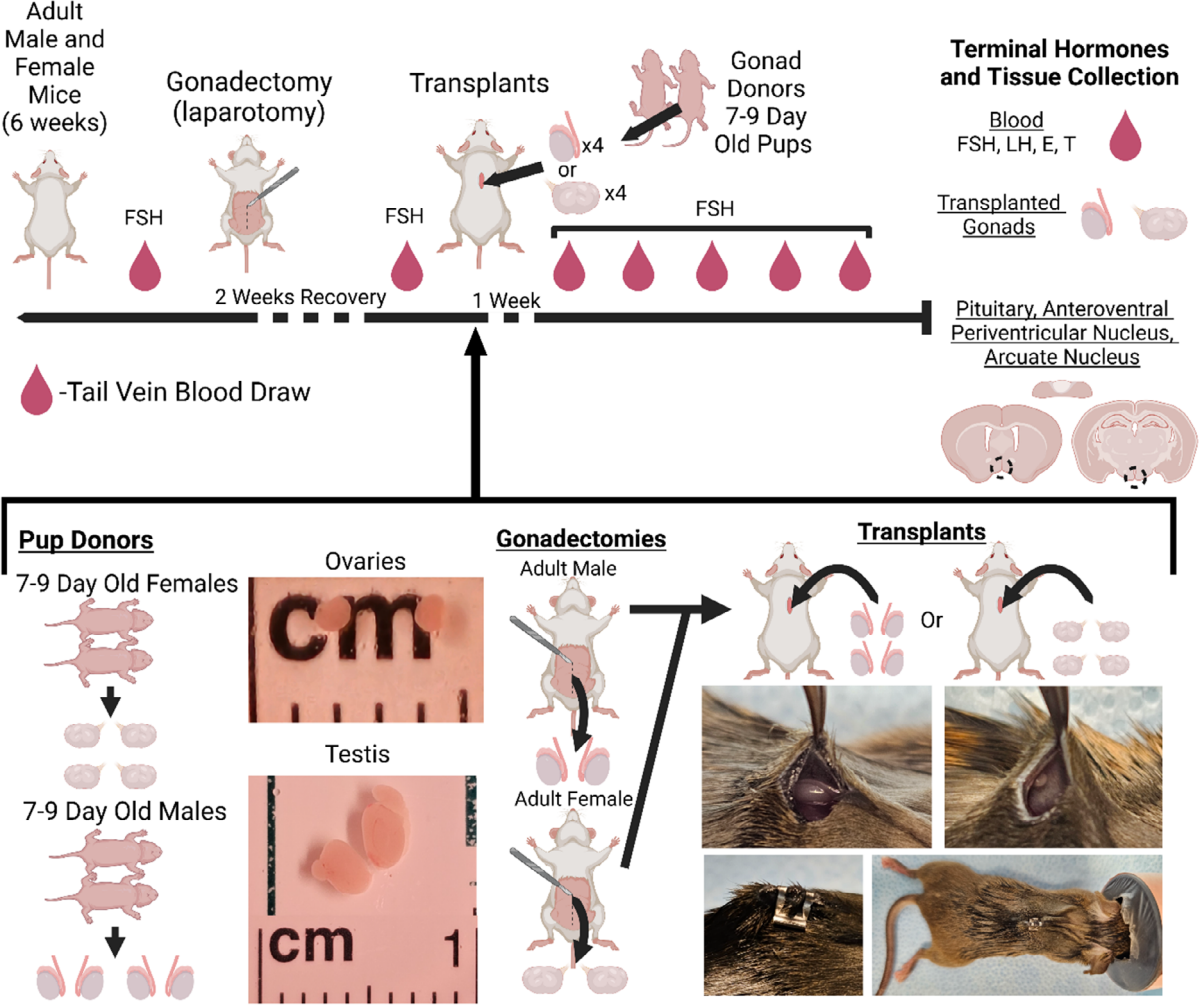
Study Design. E = Estradiol, T = Testosterone, FSH = Follicle stimulating hormone, LH = Luteinizing hormone. Created using Biorender.com.

Rodent models are being developed to inform and improve many gender‐affirming treatments [31, 32, 33, 34, 35, 36, 37, 38]. Many functions of the mammalian HPG are conserved across species, like the location and function of the hypothalamic portion of the axis. In both humans and mice, a hypothalamic region anterior and another region posterior to the optic chiasm are associated with the HPG. Both the anterior portion, the anteroventral periventricular (AVPV) nucleus, and the posterior portion, the arcuate nucleus (ARC), respond to circulating steroid hormone levels by altering the signals they send to gonadotropin‐releasing hormone (GnRH) neurons. The GnRH neurons are not concentrated into a hypothalamic nucleus, but their signals alter pituitary activity to maintain steroid hormone homeostasis and reproductive function [25]. Like in human GAHT patients, the HPG axis of GAHT‐treated mice responds to circulating T and E levels regardless of the animals’ sex [33, 34], suggesting they may be used to explore different‐sex gonad transplant integration into a novel HPG [25]. In rodents, hypothalamic and pituitary sex differences are considered organized prior to adulthood [39, 40]; however, new neurocircuitry to maintain gonad‐specific HPG functions is integrated throughout adulthood [41]. Since sex‐dependent hormonal cues are known to mediate the integration of adult‐born cells necessary for sex differences in HPG function [41], it is possible that the circulating hormonal milieu produced by transplanted gonads can do the same. Here, we asked whether different‐sex gonad transplants can be functionally integrated into the adult mouse HPG axis after a puberty driven by natal gonad type (see Figure 1 for study plan). Our objective was to determine the feasibility of different‐sex gonad transplants in adult mice and whether mice could offer a useful model for further investigations of different‐sex gonad transplants and gonad‐dependent sex differences.

## 2 Methods

### 2.1 Animals

Adult male and female mice (n = 8/group) of the B6CBAF1/J (Jackson Laboratories) strain were co‐housed five in a cage (L:D, 12:12) and provided food and water ad libitum throughout the experiment. Donor B6CBAF1/J pups (PD 6‐9) were generated from our colony of C57BL/6J and CBA/J breeding pairs (Jackson Laboratories), held under the same conditions. These MHC‐matched F1 donors are genetically identical to the mice purchased directly from Jackson Laboratories and are routinely used to avoid immunological issues when transplanting tissue [42]. All animal procedures were approved by the Institutional Animal Care & Use Committee at the University of Michigan (PRO00009635).

### 2.2 Animal Sex Nomenclature

To ensure accurate and inclusive biological discussions, particularly in studies tied to the needs of TNG individuals [43], it is necessary to address the context‐dependent nature of sex categorization [44]. For this study, ‘female’ and ‘male’ experimental mice were defined by the gonads present at the time of their gonadectomy: animals with ovaries were defined as ‘female’ and animals with testes were defined as ‘male’. Donor ‘sex’ was similarly defined by the gonads present at the time of transplanting. Accordingly, our use of the adjectives ‘same‐sex’ and ‘different‐sex’ to describe transplanted gonads refers to this gonad‐dependent characterization of sex. Differences are examined and discussed between four groups: 1) Female mice that received testes transplants (different‐sex testes transplants), 2) Female mice that received ovary transplants (same‐sex ovary transplants), 3) Male mice that received testes transplants (same‐sex testes transplants), and 4) Male mice that received ovary transplants (different‐sex ovary transplants).

### 2.3 Blood Collection and Surgical Procedures

Adult animals were left undisturbed for one week, then blood serum was collected from the tail veins the next week to measure follicle‐stimulating hormone (FSH) levels. The week after blood collection, animals were given a pre‐surgery analgesia (5 mg/kg carprofen), anesthetized (2% isoflurane, inhaled), and gonadectomized via laparotomy. Animals received a second carprofen (5 mg/kg) injection 24 h later, then were given two weeks to recover before collecting a blood sample to measure FSH. One week later, cages were randomly assigned to receive transplanted gonads from two different‐sex or same‐sex donor pups, for a total of four transplanted gonads (Figure 1). Young donors were used to maximize the number of implanted primordial/primary follicles and stem Leydig cells per unit volume of the tissue. Ovaries from 6‐9‐day‐old mice contain mostly primordial and a small fraction of activated primary and secondary follicles, while ovaries from adult mice contain corpus lutea and antral follicles [45]. Furthermore, human ovarian cortex would be used in a clinical setting and, like prepubertal mouse ovaries, would contain mostly primordial follicles. Similarly, testes from 6‐9 days old mice contain mostly stem Leydig cells [46], and pre‐pubertal tissue or stem Leydig cells are used for human and non‐human primate same‐sex testis transplants [19, 20, 21]. For transplant surgeries, donor gonads were removed immediately before anesthetizing experimental animals. Pups were rapidly decapitated, then their gonads were removed and placed in warm Leibovitz L‐15 media (∼36°C, Gibco, Thermo Scientific). For experimental animals, the previously described pre‐surgery and anesthetizing methods were performed. Scissors were used to make a small incision to the left of the midline, then to create a subcutaneous pocket where all donor gonads were placed. Wound clips (Braintree Scientific) were used to close the surgical site (Figure 1). After a week of recovery, 5 weekly tail vein blood draws were taken to measure FSH, followed by terminal cardiac blood collected on week 6 to analyze E, T, FSH, and luteinizing hormone (LH).

### 2.4 Terminal Measures

Animals were weighed and tissues sensitive to T or E levels measured, including clitoral area and uterine and seminal vesicle weight. Transplants were removed, imaged, and a portion was placed in Bouin's fixative. The brain was removed, and the pituitary placed in RNA protect (Qiagen). Brain tissue collection took place in sterile saline chilled over blue ice using sterile materials cleaned with RNAse Away (Thermo Scientific) per manufacturer instructions. The brain was placed in a brain matrix (Electron Microscopy), then a razor blade was inserted directly posterior to the optic chiasm. Two more razor blades were placed 1 and 2 mm anterior to the first, then a fourth, 2 mm posterior to the first. The anterior and posterior brain slices were placed on a slide, then a blunt hypodermic needle was used to biopsy the AVPV and ARC before placing them in RNA protect.

### 2.5 Hormone Analysis

Serum samples were kept at −20 °C before being shipped to the Ligand Assay and Analysis Core Facility, University of Virginia Center for Research in Reproduction. Sensitivity for each assay was as follows: Follicle Stimulating Hormone (sensitivity: 3 ng/mL, inter‐assay CV 9.4%; In house radioimmunoassay [47]), Testosterone (sensitivity: 10 ng/dL, inter‐assay CV 9.6%; IBL ELISA, Minneapolis, MN), Estradiol (sensitivity: 5 pg/mL, inter‐assay CV 10.2%; ALPCO ELISA, Salem, NH), Luteinizing Hormone (sensitivity: 0.04 ng/mL, inter‐assay CV 6%; In house radioimmunoassay [48]).

### 2.6 Histology

After 24 h of Bouin's fixation, gonad transplant portions were washed in ethanol dilutions, stored in 70% ethanol, and then embedded in paraffin blocks by the University of Michigan Dental School Histology Core. Transplant tissue, potentially containing multiple transplanted gonads, was sectioned serially at 5 µm, 5 sections to a slide. Every other slide was stained with hematoxylin (Epredia, Kalamazoo, MI) and eosin (Ricca Chemical, Arlington, TX; H&E), then imaged at 5X. Morphological features were evaluated by tracking structures through Z‐stacks using ImageJ, including seminiferous tubules, follicles, and corpora lutea (CL).

### 2.7 Immunohistochemistry

Leydig cells and CLs were identified in H&E‐stained testicular and ovarian tissue, respectively, and then adjacent sections were used to visualize LH receptor location using a previously published paradigm [49]. One section from one slide was stained for each animal. All procedures were performed at RT and in tris‐buffered saline (TBS) unless noted otherwise. Tissue mounted on slides was deparaffinized with xylenes, dehydrated, blocked with 0.3% hydrogen peroxide, and incubated in 0.1 m citrate buffer (pH = 6.0, Thermo Fisher, AAJ63950AP) for 20 min at 90°C, then cooled to RT for 20 min. Tissue was permeabilized and blocked with 10% normal goat serum (NGS, Abcam, G9023) in TBS with Triton for 1 h. Sections were left overnight at 4°C with rabbit anti‐LHCGR (Bioss, 1:250, bs‐0984R) in 1% NGS, then incubated with biotinylated goat anti‐rabbit secondary (Abcam, AB64256) for 1 h. Following streptavidin horseradish peroxidase (Abcam, AB64269), the sections were incubated in the DAB reaction (Abcam, AB64238) for 8 min before being counterstained with hematoxylin.

### 2.8 RNA Extraction and qPCR

A RNeasy Kit (Qiagen) was used to extract RNA from brain and pituitary tissue following the manufacturer's procedures. RNA concentrations were measured using a NanoDrop (Thermo Fisher) before converting equal amounts to cDNA using an iScript cDNA Synthesis Kit (Bio‐Rad, Hercules, CA). Target genes included *Esr1* and *Ar* for all tissues, *Kiss1, Gpr54, and Pgr* for the AVPV, *Kiss1, Gpr54, Pdyn, Tac2, and Npy* for the ARC, and *Cga, Fshb, Lhb, and Gnrhr* for the pituitary. *Fshb* Housekeeping genes tested included *Gapdh, Ppia, Rpl37*, and *Sdha* (see Table S1 for primers; Integrated DNA Technologies, Coralville, IA). *Ppia* was chosen as the housekeeping gene for the ARC and AVPV, and *Gapdh* for the pituitary. An iTaq Universal SYBR green Supermix (Bio‐Rad) was used for reactions that were run using a roto‐gene Q (Qiagen) system. LinRegPCR software [50] was used to calculate fold change values.

### 2.9 Statistical Analysis

Planned comparisons were used to analyze differences in pre‐ and post‐gonadectomy FSH levels; pre‐gonadectomy differences between sexes, within‐sex levels before and after gonadectomy, and the differences in FSH levels pre‐/post‐gonadectomy between sexes were analyzed using unpaired Mann‐Whitney or paired Wilcoxon tests (n = 7‐8/group; alpha = 0.017, alpha split for multiple tests). Normality for all other analyses was determined using the Shapiro‐Wilk test. Uterine and seminal vesicle weights and clitoral areas were compared with t‐tests (n = 7‐8/group; alpha = 0.05). A two‐way ANOVA with animal sex and gonad type as factors was performed for all other measures, followed by post hoc pairwise comparisons adjusted for multiple comparisons using Tukey's method, to investigate significant main effects or interactions (n = 5‐8/group; alpha = 0.05). Data for 2‐way ANOVA analysis without normal distributions were log transformed in Graphpad/PRISM before analysis. Outliers were visually assessed and removed from analysis if 1.5 times the interquartile range above the third or below the first quartile (see Tables S3 and S4). Data presented in results are Means±*SD* and Cohen's *d* effect sizes are included for each analysis. See figures or Supplementary Tables S3 and S4 for additional sample size information.

## 3 Results

### 3.1 Terminal Gonadotropin and Steroid Hormone Levels are Gonad‐Dependent

**FIGURE 2:**
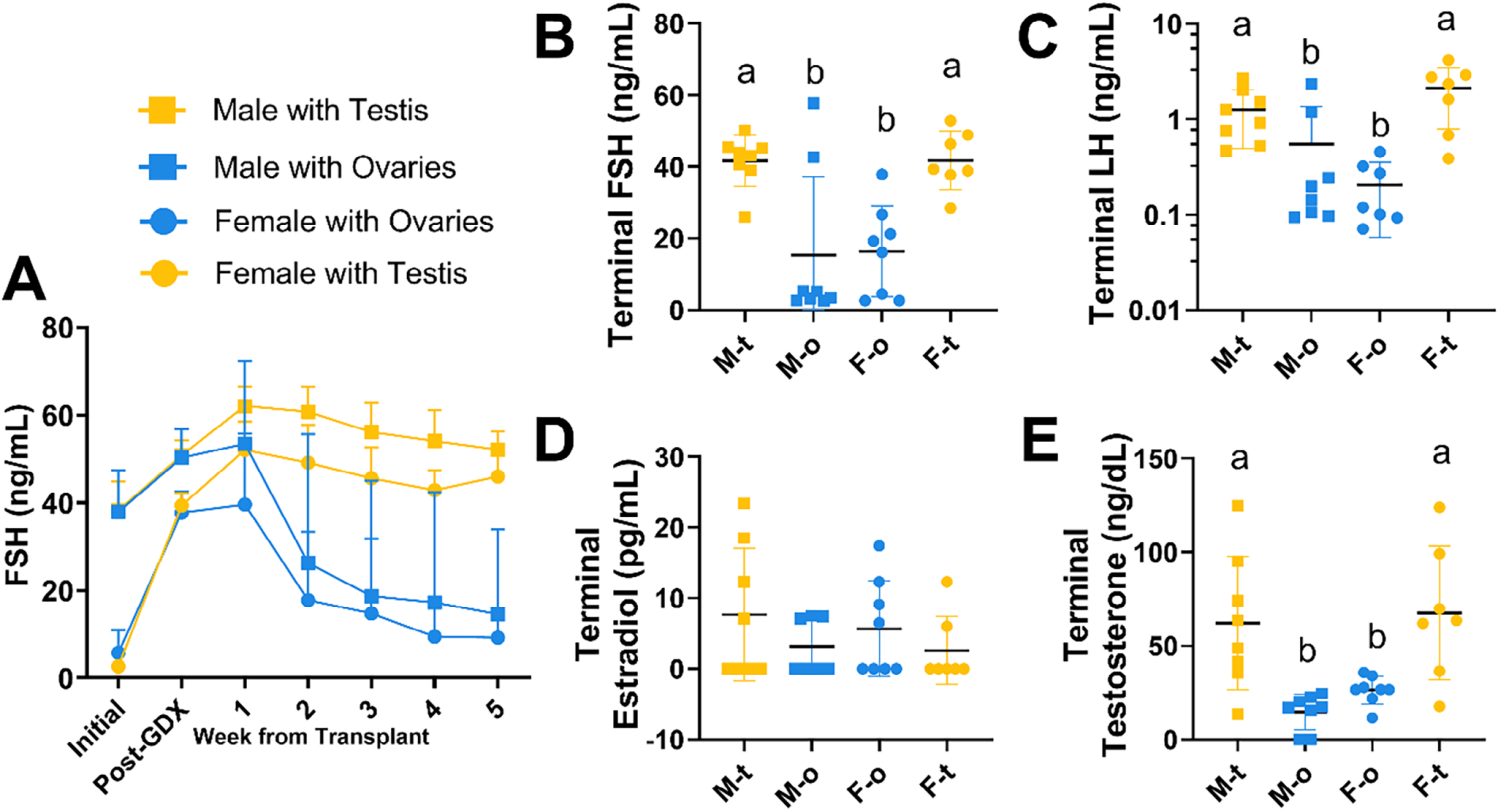
Weekly and terminal hormone levels. A) Follicle stimulating hormone (FSH) levels were higher in males compared with females pre‐gonadectomy (*p*<0.0001; Mann‐Whitney test). FSH was elevated in both males (*p*=0.0002; Wilcoxon test) and females (*p*<0.0001) post‐gonadectomy then the average starts lowering two weeks after gonad transplants were performed. B) Terminal FSH levels were highest in animals with testes. C) Likewise, terminal luteinizing hormone was highest in animals with testes. D) Terminal estradiol levels were not gonad‐ or sex‐dependent while E) terminal testosterone was higher in animals with testes compared to those with ovaries. Two‐way ANOVAs with Tukey's tests used for B‐E. Different letters are statistically different, *p*<0.05. Sample sizes: M‐t n=8, M‐o n=8, F‐o n=8, F‐t n=7.

FSH levels are higher pre‐gonadectomy in male than female mice (See ‘*Sex Nomenclature’* for definitions of ‘male’ and ‘female’; Figure 2A; See Table S2 for hormone Means ± *SD*; see Tables S3 and S4 for individual values; *p*<0.0001, *d* = 3.8). Following gonadectomy, an increase in circulating FSH was evident in males and females (Males: p = 0.0002, *d* = 1.3; Females: p<0.0001, *d* = 6.3), but the increase was greater in females (*p*<0.0001, *d* = 2.7, Pre: 4.2 ± 2.7 ng/mL FSH, Post: 38.6 ± 3.8, Difference: 34.4 ± 3.3) compared with males (Pre: 38.1 ± 8 ng/mL FSH, Post: 50.6 ± 5, Difference: 12.46 ± 6.5). Terminal FSH and LH levels were higher in animals with testes (Figure 2B,C; FSH *p*<0.0001, *d* = 1.3; LH *p* = 0.0003, *d* = 1.1) but independent of sex (FSH *p* = 0.9, *d* = 0.03; LH *p* = 0.07, *d* = 0.2). Terminal E levels were independent of both gonad type (Figure 2D, *p* = 0.4, *d* = 0.08) and sex (*p* = 0.1, *d* = 0.1). Mice with testes had higher terminal T levels than those with ovaries (Figure 2E, *p*<0.0001, *d* = 1.2), independent of sex (*p* = 0.7, *d* = 0.2).

### 3.2 Anatomical Changes Are Gonad‐Dependent

**FIGURE 3:**
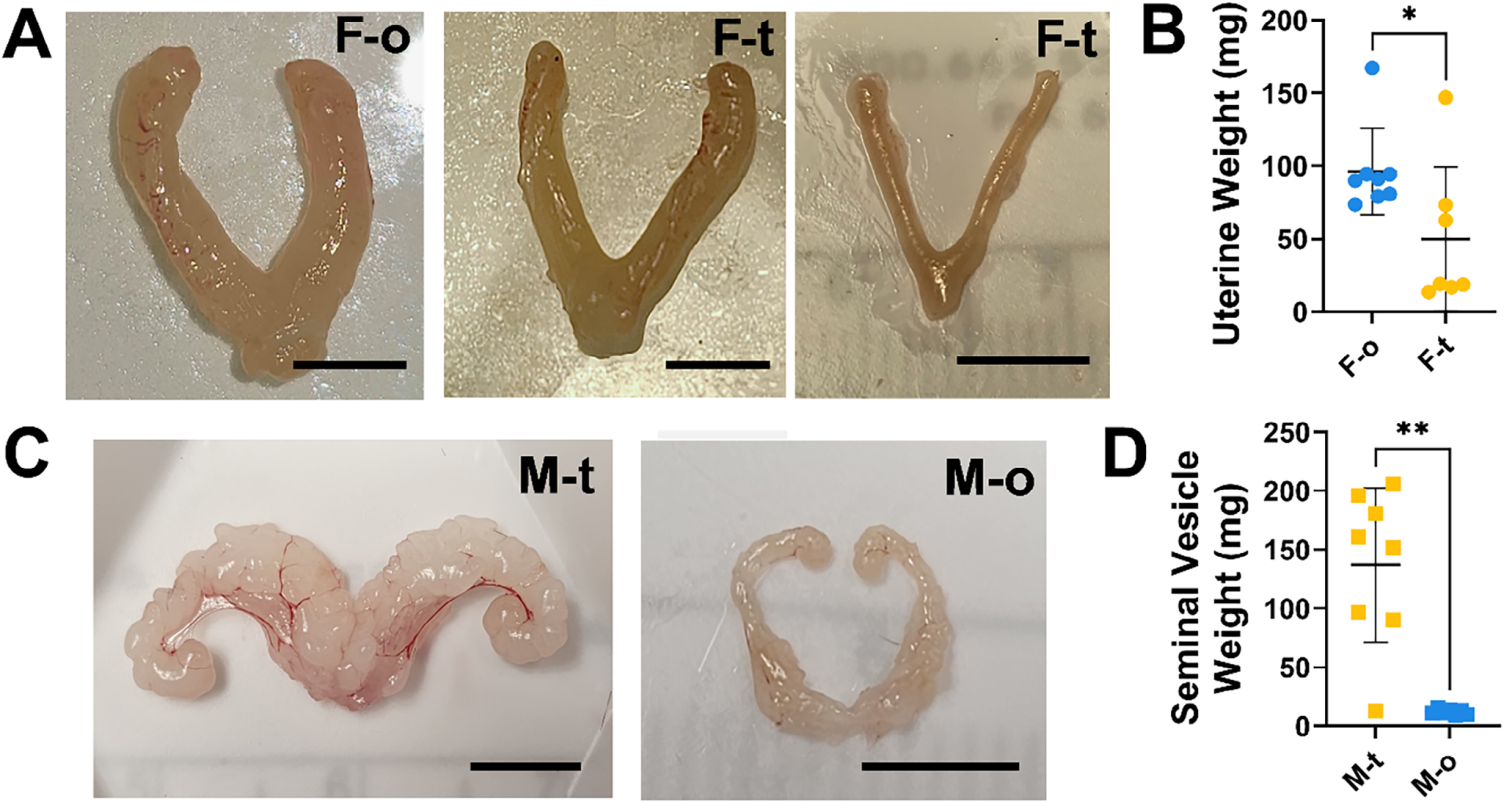
Reproductive Anatomy. A) Uterus B) weight was highest in females given ovary (F‐o, n=8) transplants and there was large variability in the size of uteri from females implanted with testes (F‐t, n=7). C) Seminal vesicles were D) heavier in males that received testes (M‐t, n=8) transplants compared with those given ovaries (M‐o, n=7). T‐tests, *p<0.05, **p<0.005.

Male mice weighed more than females (see Tables S3 and S4 for individual values, *p*<0.0001, *d* = 1.5), regardless of gonad type (*p* = 0.11, *d* = 0.08). Female mice with testes had larger clitorises than those with ovaries (see Tables S3 and S4 for individual values, *p* = 0.004, *d* = 1.7) while female mice with ovaries had heavier uteri than those with testes (Figure 3A,B, *p* = 0.014, *d* = 1.1). Male mice with testes had heavier seminal vesicles than males with ovaries (Figure 3C,D; *p* = 0.002, *d* = 2.7).

### 3.3 Testis Transplant Anatomy and Histology Indicate Successful HPG Integration

**FIGURE 4:**
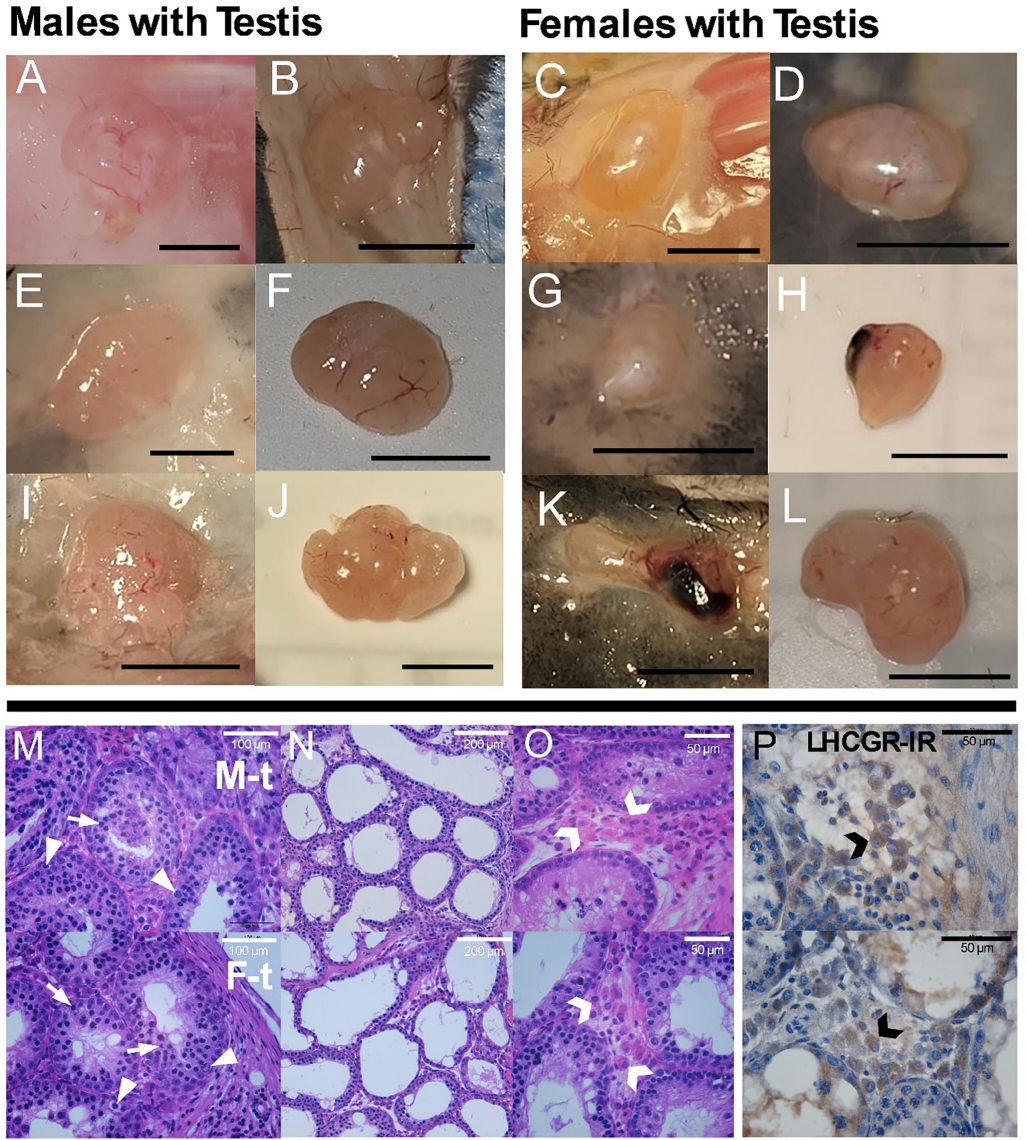
Representative images of transplanted testes anatomy and histology. A–L) testes transplants from male (M‐t) and female (F‐t) animals display variable growth, vasculature and morphology. Transplants were attached to the epidermal layer or embedded in muscle/adipose tissue. There is variability in overall transplant size and the presence of irregular/smooth margins or bloody portions, which may indicate differences in the number of donor gonads that survived and grew. M) Histology indicates seminiferous tubules (hematoxylin and eosin) are present in all transplants with clear evidence of spermatogonia (arrowhead) and primary spermatocytes (arrow). N) Many transplants contained seminiferous tubules with dilated lumens and thinner tubes. O) Leydig cells (chevron) were identified by their location between seminiferous tubules, shape, and P) expression of the luteinizing hormone receptor (Brown LHCGR‐IR, blue hematoxylin counterstain).

Transplanted testes fused to form connected structures, likely containing multiple transplanted gonads (see Table S3; Figure 4A,B,E,F,I,J,L), and embedded in muscular/adipose (Figure 4A,C) and epidermal tissue (Figure 4B,D,E,G,K). Before removal, vasculature was visible, surrounding testis transplants (Figure 4A,B,C,D,G,I,K). Most testis transplants displayed irregular margins but several transplants removed from female hosts were smooth (Figure 4C,D) or had what appeared to be unhealthy tissue (Figure 4H,K). Histology revealed structures resembling seminiferous tubules were present in all testis transplants (Figure 4). All testis transplants, aside from one taken from a female, had seminiferous tubules with varying degrees of spermatogenesis up to the spermatocyte stage (Figure 4M). These tubules displayed spermatogonia (Arrowheads), spermatocytes (Arrows), and cytoplasmic processes from Sertoli cells extending into their lumens (Figure 4M). Some structures resembling seminiferous tubules lacked these cytoplasmic processes and had empty lumens (Figure 4N). No testis transplants had recognizable spermatids. Leydig cells (Chevron) were identified in all testis transplants taken from male and female mice, based on their morphology, location between the seminiferous tubules (Figure 4O), and the presence of LH receptors (Figure 4P).

### 3.4 Ovary Transplant Anatomy and Histology Indicate Successful HPG Integration

**FIGURE 5:**
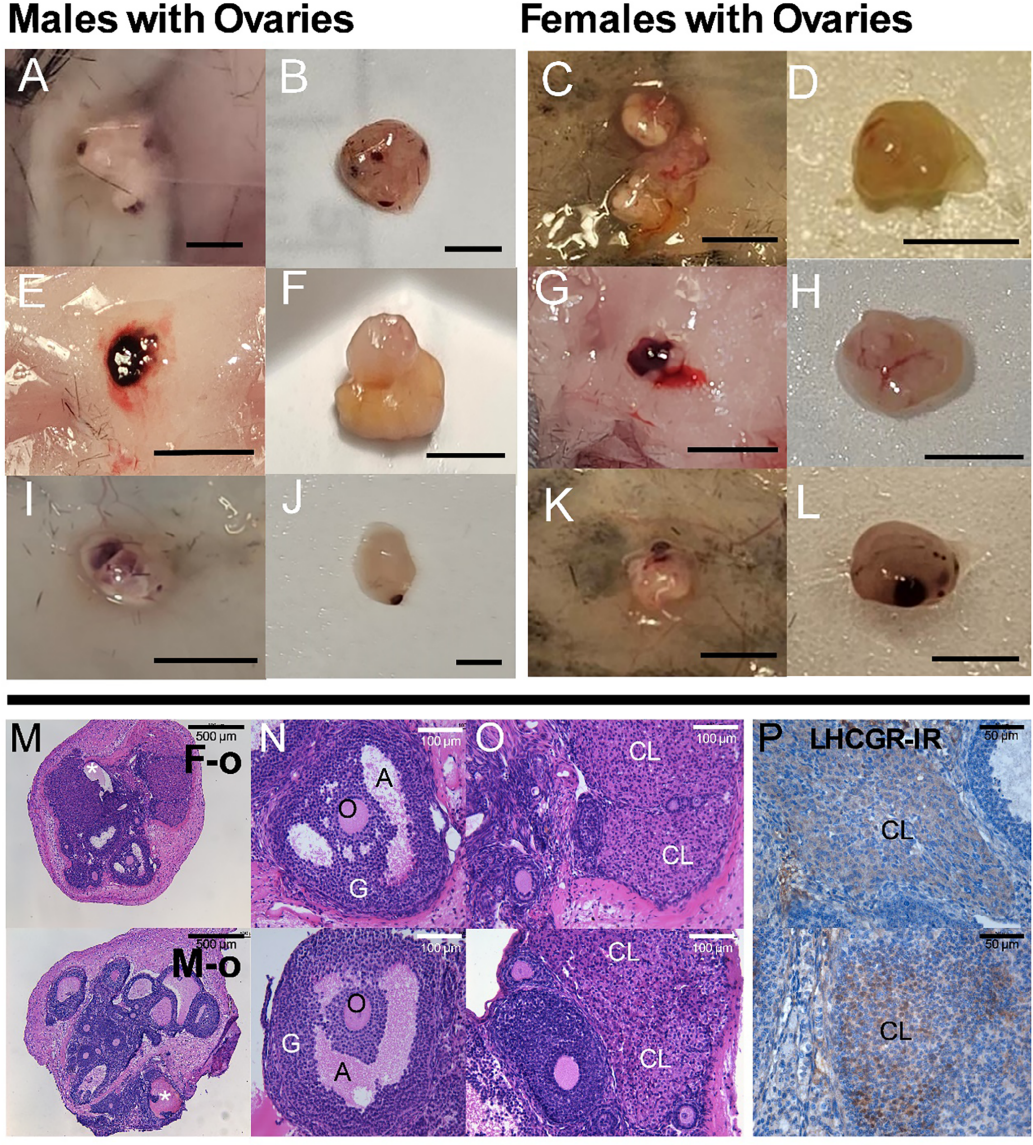
Representative images of transplanted ovary anatomy and histology. A–L) Ovary transplants from males (M‐o) and females (F‐o) show growth and vascularization. Many transplants taken from males, and a few from females, had small blood spots or larger blood patches, potentially sites of un‐ovulated cystic follicles (asterisk). Structures resembling fluid‐filled antral follicles and yellowed luteal tissue were present in the ovaries of males and females. M) Ovarian histology (hematoxylin and eosin) indicates the presence of N) antral follicles (A) with intact antrum (A), oocytes (O) and granulosa (G) cells, and O) corpora lutea (CL), P) which express the luteinizing hormone receptor (Brown LHCGR‐IR, blue hematoxylin).

Many ovarian transplants fused to form a structure containing multiple transplanted gonads in female and male mice (see Table S4; Figure 5A,B,D,F). Other ovarian transplants from female and male mice had multiple discrete portions that were, nonetheless, in close apposition (Figure 5C,I). Additionally, most ovary transplants had irregular margins (Figure 5A,B,D,F,H) with some protruding structures having a bloody appearance (Figure 5) and others resembling the opaque fluid‐filled antrum of antral follicles (Figure 5B,D,F,H) or yellowed, potentially luteal, tissue (Figure 5A,C,F,K). Most transplanted ovaries from males and some from females had small bloody protrusions or patches (Figure 5E,G,I,J,K,L). Histological analysis of ovary transplants (Figure 5M) uncovered primary, secondary and tertiary follicles in all transplants, and most also had antral (Figure 5N) and atretic antral follicles, characterized by direct contact between oocyte (O) and antral fluid (A). However, one male's ovary transplant contained only pre‐antral follicles and cystic antral follicles. Three transplants taken from female mice had cystic antral follicles while these blood‐filled structures were present in all ovaries taken from males (Figure 5M, asterisk). Corpora lutea (CL), identified by their histological structure (Figure 5O) and LHCGR receptor expression (Figure 5P), were present in all ovary transplants taken from female mice and in two taken from male mice.

### 3.5 Hypothalamic and Pituitary Gene Expression is Sex‐ and Gonad‐Dependent

**FIGURE 6:**
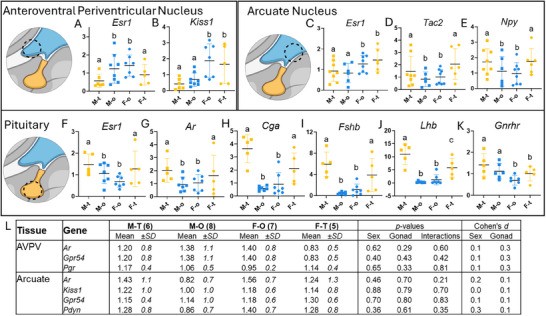
Brain and pituitary gene expression. AA) Animals with ovaries had higher *Esr1* expression in their anteroventral periventricular nucleus (AVPV) than those that received testes, regardless of sex. B) Conversely, *Kiss1* expression was highest in female animals compared with males, regardless of gonad type. C) Female animals had higher *Esr1* expression in their ARC than males, regardless of gonad type. Further, ARC). D) *Tac2* and E) *Npy* expression was highest in animals that received testes, regardless of sex. F‐I) Animals with testes had higher pituitary *Ar, Esr1, Cga*, and *Fshb* expression than those with ovaries, regardless of gonad type. J) Male animals with testes had the highest *Lhb* levels, followed by females with testes, while animals with ovaries had the lowest *Lhb* expression levels. K) Male animals had higher *Gnrhr* expression in their pituitary than females, regardless of gonad. L) No significant differences or large effects in AVPV *Ar, Gpr54*, and *Pgr*, nor ARC *Ar, Kiss1, Gpr54*, and *Pdyn* expression were seen. Two‐way ANOVAs with Tukey's tests, different letters are statistically different, *p*<0.05. AVPV sample sizes: males with testis (M‐t) n=6, males with ovaries (M‐o) n=8, Females with ovaries (F‐o) n=7, Females with testis (F‐t) n=5. ARC sample sizes: M‐t n=8, M‐o n=6, F‐o n=8, F‐t n=6. Pituitary sample sizes: M‐t n=5, M‐o n=7, F‐o n=7, F‐t n=5.

Several genes across the HPG showed significant differences based on sex and/or gonad type (Figure 6). Within the AVPV, *Esr1* expression was highest in animals with ovaries compared to those with testes (Figure 6A; *p* = 0.03, *d* = 0.7) while AVPV *Kiss1* levels were higher in females than males (Figure 6B; *p*<0.001, *d* = 1). There were no significant main effects of sex or gonad nor their interaction for the expression of AVPV *Ar*, *Gpr54*, and *Pgr* (Figure 6L). In the ARC, *Esr1* was highest in female animals compared to males (Figure 6C; *p* = 0.01, *d* = 0.7). Conversely, ARC *Tac2* (Figure 6D; *p* = 0.03, *d* = 0.6) and *Npy* (*p* = 0.04, *d* = 0.6) expression levels were higher in animals with testes than those with ovaries, regardless of sex. ARC genes without significant differences included *Ar*, *Kiss1*, *Gpr54*, and *Pdyn* (Figure 6L). Finally, pituitary expression of *Esr1* (Figure 6F; *p* = 0.04, *d* = 0.6), *Ar* (Figure 6G; *p* = 0.05, *d* = 0.6), *Cga*,(Figure 6H; *p* = <0.0001, *d* = 1.4), and *Fshb* (Figure 6I; *p* = 0.0001, *d* = 1.2) levels were highest in animals with testes and independent of sex. A significant interaction between gonad and sex was seen when comparing means for *Lhb* (Figure 6J; *p* = 0.01): males with testes had higher *Lhb* than females with testes (*p* = 0.02, *d* = 1.4), while males with ovaries had lower levels than males (*p*<0.0001, *d* = 3.8) or females (*p*<0.01, *d* = 2.1) with testes. Females with ovaries also had lower *Lhb* levels than males (*p*<0.0001, *d* = 3.2) or females (*p* = 0.03, *d* = 1.6) with testes. *Lhb* did not significantly differ between females and males with ovaries (*p* = 0.9, *d* = 0.6). Pituitary *Gnrhr* expression was higher in males compared with females (Figure 6K; *p* = 0.02, *d* = 0.8) but independent of gonad type (*p* = 0.07, *d* = 0.6).

## 4 Discussion

Here, we show that the adult mouse hypothalamus and pituitary can integrate different‐sex gonads into the host HPG axis. Multiple outcomes supported the incorporation of testis transplants. Though pre‐gonadectomy male mice (See *Sex Nomenclature* for characterization of ‘male’ and ‘female’) initially had higher FSH than females, gonadectomy increased FSH levels, removing this sex difference. Testes typically rely on high FSH to develop during puberty [51, 52], so the high circulating FSH of gonadectomized animals likely supported the maturation of testes transplants. Mature testis transplants could then respond to LH by producing T [53], which provided negative feedback to the hypothalamus and pituitary to maintain HPG homeostasis [54]. Indeed, following transplantation of testes, FSH in males and females lowered to levels comparable to those of pre‐gonadectomy males, suggesting reestablished negative feedback. Terminal steroid measures provided direct evidence of elevated T produced and secreted from the transplanted gonads. Further, terminal anatomy suggested sustained T elevation as androgen‐sensitive tissues followed expected patterns for male [55, 56] and female mice [34] with high T—males with testes had larger seminal vesicles than those with ovaries, and females with testes displayed clitoromegaly. Histology revealed the presence of mature Sertoli and Leydig cells but limited spermatogenesis in testes transplants [57]. These elements are absent in testes from PD 6‐9 pups [46, 51, 58] and must have developed following transplantation. Leydig cells were located between seminiferous tubules, expressed receptors for LH, and were the likely source for elevated T in animals with testis transplants [53, 59]. Though gametogenesis was not the focus of this study, the varying levels of sperm maturation seen in transplants required the presence of T [60, 61, 62]. Many seminiferous tubules had mature Sertoli cells, spermatogonia, and spermatocytes, but no spermatids were seen. Given that spermatogenesis is a 30‐day cycle and requires Sertoli and Leydig cells to mature [63, 64], transplants may not have had sufficient time to complete spermatogenesis. Alternatively, processes may have inhibited spermatogenesis. Indeed, some tubules with spermatogonia had dilated lumens and lacked recognizable Sertoli cells. This morphology is similar to testes, where spermatogenesis is prevented by altered physiology or disease states [65, 66]. Notably, an increase in luminal pressure can lead to this morphology [67, 68], and encapsulation of transplanted gonads by host tissues may have increased pressure by blocking outflow from seminiferous tubules. Overall, we found that testis transplants addressed the needs of a novel method for providing gender‐affirming hormone therapy; testis transplants in female mice matured, began producing T in response to host gonadotropins, and provided negative feedback to the hypothalamus and pituitary regardless of sex.

Functional integration of ovarian transplants was also supported by several findings. As before, gonadectomy elevated FSH levels in male and female animals prior to implantation and removed the sex difference. The ovaries respond to FSH by producing E, which communicates with the hypothalamus and pituitary in multiple ways throughout the gonad cycle [69, 70, 71] to modulate gonadotropin release [22, 72, 73]. Ovary transplantation decreased FSH in males and females to levels comparable to females before gonadectomy. Further, female mice given ovaries had heavier uteri, which are highly responsive to E [74, 75]. Interestingly, ovary transplants had no effect on terminal E levels in males or females. This may be due to the relatively low sensitivity of the E immunoassay used [76, 77]. Indeed, though we have previously found dose‐dependent effects of E treatment on anatomy in male mice, this assay could not identify dose‐dependent effects on circulating estradiol [33]. Although elevated E has been identified in mice given ovarian transplants, this was through comparisons with ovariectomized animals [78], while our comparisons were between animals with ovaries or testes. It will be critical to use more sensitive assays [79] in future studies. It may also be necessary to take multiple serum samples to measure E over time in mice with ovarian transplants, which may cycle between periods of high and low E. Despite our inability to detect elevated E, we observed several E‐dependent processes histologically. Ovaries taken from PD 6‐9 pups contain mostly primordial and primary follicles [45], which require both FSH and E to fully mature [80]. The appearance of primary, secondary, and antral follicles in ovary transplants indicates folliculogenesis occurred after transplantation. This process not only relies on E but also involves E secretion from granulosa cells [70, 81]. Overall, our findings suggest that ovary transplants meet the needs of E‐GAHT; ovaries transplanted into male mice matured through E‐dependent processes and provided negative feedback to the hypothalamus and pituitary. In addition to E‐producing follicles, transplanted ovaries from females and two from males had CLs. These LH‐sensitive structures form from ovulated follicles and produce progesterone [82]. Though not included in evidence‐based guidelines for E‐GAHT, progesterone is a component of E‐GAHT for some patients. Some argue that sufficient evidence supports the addition of progesterone [83] and, should guidelines change, this method may be capable of meeting that need. An obvious barrier to using this mouse model is the rarity of progesterone‐producing CLs in ovaries transplanted into male mice (n = 2). Critically, our histological analysis may have failed to identify all CLs formed in the ovarian transplants, as only half of the transplanted tissue was examined. In future studies, analysis of whole transplants will be important, allowing comparisons between potential subpopulations in males based on CL presence. The appearance of blood‐filled cystic antral follicles in all ovary transplants taken from males, and many from females, is indicative of fully developed antral follicles that did not or could not ovulate [84]. Cystic follicles may have matured shortly after transplantation in response to high FSH or even mechanical stimulation [70, 80, 85], then reached maturity before fully integrating their positive feedback into the HPG. Once ovarian transplants could provide a signal sufficient to produce an LH surge [86], antral follicles may have ovulated and formed CL. Given that ovary transplant development appears to proceed differently in male or female animals, long‐term studies should examine multiple cohorts at different timepoints. Indeed, males with CLs also had cystic follicles, so delaying terminal tissue collection and increasing group number may help bolster the number of CL‐producing males. As this study lacked continuous monitoring of LH secretions, and the potential for follicles to undergo premature luteinization in the absence of an LH surge exists [87], we cannot definitively associate CL formation in our animals with the presence of an LH surge. To fully characterize the steroidogenic capacity and integration of different‐sex ovary transplants (See *Sex Nomenclature* for characterization of ‘same‐sex’ and ‘different‐sex’ transplants), it will be essential to examine relationships between CL formation, LH surges, and the local production of progesterone in future studies.

Although the adult human and primate HPG axis appears to possess sufficient plasticity to support different‐sex gonad cycles [26, 27, 30], it was assumed that the HPG axis of rodents would be permanently organized by adulthood [88, 89, 90, 91, 92]. Our findings contrast these early assertions of either the functional limits or immutability of HPG sex differences in mice. Given gonad‐dependent gonadotropin levels, we probed the expression of upstream HPG signaling genes. Genes that produce receptors or neurotransmitters related to gonadotropin release were investigated in two hypothalamic regions. The ARC contains a population of neurons that receive negative feedback from steroid hormones and express the neurotransmitters kisspeptin (KISS), neurokinin B (NKB), dynorphin, and neuropeptide y (NPY), called KNDy neurons. These neurotransmitters modulate the activity of both KNDy and gonadotropin‐releasing hormone (GnRH) neurons to maintain HPG homeostasis [22, 73]. Testis transplants increased *Tac2* (NKB) and *Npy* (NPY) mRNA, independent of sex. Increased NKB or NPY signaling may have elevated gonadotropin levels in animals with testes, as genetic knockouts of NKB receptors are associated with gonadotropin deficiencies in humans and mice [93, 94], and NPY activates GnRH neurons [95]. Elevated NKB or NPY in animals with testes may have facilitated downstream changes in circulating gonadotropins [95, 96]. Notably, the ARC is thought to produce LH pulses in males and females [97, 98]. These changes may be necessary to ensure similar pulse generation under varying hormonal conditions. Indeed, the ARC functions similarly in males and females [97] despite variability in Esr1 expression [99]. Interestingly, the sex difference in ARC *Esr1* expression was independent of gonad type. Notably, ARC *Esr1* is also involved with non‐HPG processes, such as calcium and energy homeostasis in bones [100], so such changes may be unrelated to gonad‐dependent HPG function. These expression patterns are also relevant to the human ARC, where *ESR1* and *TAC3* (NKB) expressing cells drive regional sex differences tied to many health outcomes [101]. Characterizing health‐related endpoints (musculoskeletal health, glucose homeostasis, inflammatory pathways, etc.) in future mouse experiments will be necessary to inform the safety of gonad transplants.

Another steroid hormone‐sensitive HPG neuron population is the kisspeptin neurons in the AVPV. These also control GnRH neuron activity but receive both positive and negative feedback from the gonads, leading to surges of LH. Two days of exogenous E treatments to mimic proestrus levels (E‐priming) is sufficient positive feedback to induce an LH surge in female rodents, but not males [92]. Critically, AVPV neurons expressing *Esr1* are essential for producing LH surges [102], and male mice typically have less AVPV *Esr1* mRNA than females [103]. These sex differences are thought to permit the production of LH surges in females [92]. Though E‐priming fails to initiate an LH surge in males [103], we saw potential evidence of LH surges via CL formation in ovaries from both males and females. Notably, E‐priming elevates AVPV *Esr1* in females but not males [92], yet we found that ovary transplants increased *Esr1* expression in the AVPV, regardless of sex. This change in *Esr1* expression may rely on several mechanisms, such as changes in gene expression or even the integration of newly divided neurons into the adult hypothalamus. Newly divided cells are integrated into the AVPV throughout adulthood, and blocking adult neurogenesis prevents the production of LH surges [41]. It is possible that established and/or newly divided AVPV cells in male mice with ovaries took on characteristics normally found in females during prolonged exposure to ovaries. That is, the hormonal environment in which cell division and development occurred may determine their function once integrated into the hypothalamus. Two days of E‐priming may be insufficient to shift AVPV function in males, but several weeks of current or newly divided cells integrating signals from transplanted ovaries might do this. Similarly, gonadectomized people assigned male at birth can produce an LH surge after E‐priming, but this proceeds differently based on previous exposure to E. Those exposed to E prior to E‐priming took only two days to produce a LH surge, while those without prior E exposure took four to five days [26]. However, if adult neurogenesis in the human hypothalamus [104] is involved, species differences may cause the integration of donor gonads to proceed differently in clinical settings, as newly divided brain cells in adult humans take longer to develop and travel farther [105]. Overall, our findings call into question the rigidity of adult mouse HPG sex differences, suggesting gonad type influences ongoing processes that can change which signals activate organized circuits. Conversely, we found expression of *Esr1* in the ARC and *Kiss1* in the AVPV is higher in females regardless of the gonad. The retention of these sex differences suggests they may be dispensable for gonad‐specific function and/or retain functional plasticity despite similar mRNA profiles.

Downstream from the hypothalamus, some mRNA expression levels in the pituitary aligned with high circulating gonadotropin levels in animals with testes. The subunits responsible for producing FSH and LH (*Fshb*, *Lhb*, and *Cga*) were highest in animals with testis transplants compared to those with ovaries. However, receptor gene expression appears to contradict gonadotropin outcomes. E can act directly on the pituitary to decrease gonadotropin release [106], yet animals with ovaries had lower pituitary *Esr1* levels and gonadotropins than animals with testes. Similarly, GnRH binding to its receptor increases gonadotropins [72, 107], and males have higher *Gnrhr* expression overall compared with females, but males with ovaries had lower gonadotropins than females with testes. This suggests feedback received by the hypothalamus is increasing gonadotropin subunit expression and circulating levels in animals with testes and/or lowering them in animals with ovaries. Though we have identified gonad‐dependent gene expression in the ARC and AVPV, GnRH neurons are the direct link between these brain regions and the pituitary [72, 95]. The diffuse distribution of these neurons could not be sampled using our methods, but characterizing changes in GnRH neurons will be essential to fully understand gonad‐dependent HPG function. When considering translation, estradiol can decrease human *GnRH* promoter activity in vitro [108], implying the integration of novel gonads could involve changes to GnRH neuron activity worth characterizing. Further, gonad differences in non‐steroidal products, like inhibins [109] and activins [110, 111], may act on and within these regions to influence gonadotropin release and/or gene expression. Overall, we identified gonad‐dependent mRNA levels that may be essential for different‐sex gonad incorporation into a novel HPG. Identifying the origins and functional significance of gonad‐dependent outcomes may help understand and improve different‐sex gonad transplants.

Beyond translation to gender‐affirming care, gonad transplants in mice expand available in vivo research methods for examining sex differences [112]. The HPG feedback circuit produces cyclic steroidal and non‐steroidal hormone levels, leading to differential activation of downstream pathways mediating innumerable measures of health [113, 114]. All gonads can secrete E and T, but hormone‐dependent sex differences are currently investigated by manipulating the main steroidal product of gonads or their receptors alone [115, 116]. Gonad‐dependent differences tied to health outcomes, like hypothalamic *Esr1* expression [117, 118, 119, 120], are obscured by the hormone replacement techniques used in sex difference research [121]. For example, AVPV *Esr1* levels are unaltered in gonadectomized males given E alone [92], but we found elevated *Esr1* levels in gonadectomized males given ovaries. Although we found females with testes had higher ARC *Tac2* mRNA than females with ovaries, gonadectomized female mice given a non‐aromatizable androgen for two weeks have lower ARC *Tac2* mRNA than untreated controls [122]. In male mice, E treatment can decrease ARC *Kiss1* expression in gonadectomized males [123], but we see no effect of ovary transplants on ARC Kiss1 compared with testes transplants. Finally, we found that animals with testes had higher *GnRHr* mRNA in their pituitary, but treating mouse gonadotrope cells with DHT does not increase *GnRHr* mRNA [124]. These data underscore significant differences between how gonads and their associated steroid hormones impact the hypothalamus and pituitary. A same‐sex‐ and different‐sex gonad transplant mouse model expands the sex characteristics available for manipulation in research, allowing basic and pre‐clinical investigators to independently examine or compare gonad‐ and steroid hormone‐dependent outcomes.

In conclusion, these data support the feasibility of different‐sex gonad transplants in adult mice and their utility as a model for investigating this potentially novel method of providing gender‐affirming hormones to TNG patients. Beyond investigating gonad transplant success, their impact on metabolism, circadian rhythms, behavior, and other health‐related outcomes may be examined in mice to identify and address undesired outcomes. Such data will be critical before performing similar manipulations and health‐related examinations in non‐human primates with an eye toward eventual translation to humans. A clinical procedure would rely on donor tissue and may require immunosuppressing or isolating methods to function [125, 126]. Our work indicates mice may be used to identify and develop immunological interventions through controlled experimentation [42, 78]. Critically, the transfer of potentially viable gametes between humans requires ethical examination and must inform conversations with potential donors. An alternative strategy could be implantation of autologous stem‐cell derived steroidogenic cells, produced through CRISPR/Cas9 manipulations and paracrine treatment of stem cells [132]. The use of autologous stem‐cell‐derived transplants could extend to the production of viable gametes, allowing individuals to produce genetic offspring. Additional procedures used by or in development for cisgender patients, such as artificial ovaries [127], uterine transplants [9], and phalloplasty [128], may expand future gender‐affirming transition options to include many aspects of reproductive function. Finally, it is essential to consider how a novel gender‐affirming hormone technology will be received by the intended users. For example, patients may opt for GAHT over a gonad transplant, and this procedure should be considered an alternative, rather than a replacement, to current options. In addition to investigating this model, researchers should seek collaborations with both transgender studies scholars [38, 43] to interrogate the implications of this technology, and TNG people [129, 130, 131], to ensure ‘sex’ nomenclature, study questions, interpretations, and products align with community needs and desires.

## Author Contributions

Study Design (DRP, JGC, MAR, VP, AS, MBM), Acquisition (DRP, EC, JGC, RKC, REK, AS), Analysis (DRP, EC, JGC, REK, MAR, AS), Interpretation (All), Manuscript Drafting (DRP), Manuscript Preparation (All).

## Funding

NIH T32 DK071212, R01 HD098233.

## Conflicts of Interest

The authors declare no conflict of interest.

## Supporting information




**Supporting file**: adbi70081‐sup‐0001‐SuppMat.docx

## Data Availability

The data that support the findings of this study are available from the corresponding author upon reasonable request.
