# Supplementary material for: Functional Integration of Different‐Sex Gonad Transplants Into the Adult Mouse Hypothalamic Pituitary Gonadal Axis
Source: Adv Biol (Weinh). 2025 Dec 7;10(1):e00316. doi: 10.1002/adbi.202500316 (PMC12798698; doi:10.1002/adbi.202500316)
Supplement: Supplementary file 1 — Supporting file: adbi70081‐sup‐0001‐SuppMat.docx [file ADBI-10-e00316-s001.docx]

Supplementary Table S1: Primers for qPCR reactions.

| **Gene** | **Forward Primer** | **Reverse Primer** |
| --- | --- | --- |
| *Ar* | CCTTGGATGGAGAACTACTCCG | TCCGTAGTGACAGCCAGAAGCT |
| *Cga* | CAGGTCCAAGAAGACAATGCTGG | TGCTACAGTGGCACTCCGTATG |
| *Esr1* | TCTGCCAAGGAGACTCGCTACT | GGTGCATTGGTTTGTAGCTGGAC |
| *Fshb* | ATACCACTTGGTGTGCGGGCTA | AGCCAGGCAATCTTACGGTCTC |
| *Fshr* | GAGGCAGATGTGTTCTCCAACC | TCGGAGACTGGGAAGATTCTGG |
| *Gnrhr* | CTCAGCATTGTCTTTGCAGGACC | ATGCCACCACTGTGGAAAGCTG |
| *Gpr54* | GGAGACTTCATGTGCAAATTCGTC | ACATACCAGCGGTCCACACTCA |
| *Kiss1* | GCTGGCAAAAGTGAAGCCTGGA | GGCATGGCGACGACCTACGAG |
| *Lhb* | ACCTTCACCACCAGCATCTGTG | GGACAGATGCGAAGCGCAGCT |
| *Lhcgr* | AATGGGACGACGCTAATCTCGC | TGAGCGTCTGAATGGACTCCAG |
| *Npy* | TACTCCGCTCTGCGACACTACA | GGCGTTTTCTGTGCTTTCCTTCA |
| *Pdyn* | CTGTGTGCAGTGAGGATTCAGG | GAGACCGTCAGGGTGAGAAAAG |
| *Pgr* | CTACTCGCTGTGCCTTACCATG | CTGGCTTTGACTCCTCAGTCCT |
| *Tac2* | TGCTTCGGAGACTCTACGACAG | GTCCCACAAAGAAGTCGTGCATG |
| *Gapdh* | CATCACTGCCACCCAGAAGACTG | ATGCCAGTGAGCTTCCCGTTCAG |
| *Ppia* | CATACAGGTCCTGGCATCTTGTC | AGACCACATGCTTGCCATCCAG |
| *Sdha* | GAGATACGCACCTGTTGCCAAG | GGTAGACGTGATCTTTCTCAGGG |
| *Rpl37* | CCTACCACCTTCAGAAGTCGAC | CTTTAGGTGCCTCATCCGACCA |

Supplementary Table S2: Average hormone levels by group. M-t=Males with testis, M-o=Males with ovaries, F-o=Females with ovaries, F-t=Females with testis, FSH=Follicle stimulating hormone, LH=Luteinizing hormone.

Supplementary Table S3: Testis transplant mouse data. GDX=gonadectomy, FSH=follicle stimulating hormone

Supplementary Table S4: Ovary transplant mouse data. GDX=gonadectomy, FSH=follicle stimulating hormone.
